# Evolution of age at primiparity in pinnipeds in the absence of the quality–quantity trade‐off in reproduction

**DOI:** 10.1002/ece3.5138

**Published:** 2019-04-15

**Authors:** Stephanie Kalberer, Eugene DeRango, Fritz Trillmich, Oliver Krüger

**Affiliations:** ^1^ Department of Animal Behaviour Bielefeld University Bielefeld Germany

**Keywords:** co‐evolution, lactation length, fitness, life history evolution, relative birth mass

## Abstract

Age at primiparity (AP) is a key life history trait which is crucial to the evolution of life history strategies. This trait is particularly interesting in pinnipeds (walrus, eared seals, and true seals), which are monotocous animals. Thus, the commonly observed trade‐off between offspring quality and quantity does not apply to this taxon. Therefore, comparative studies on the evolution of AP might shed light on other important evolutionary correlates when litter size is fixed. Using phylogenetic generalized least squares analyses, we found a strong negative and robust correlation between relative birth mass (mean pup birth mass as a proportion of mean adult female mass) and AP. Rather than trading‐off an early start of reproduction with light relative offspring mass, this result suggests that pinnipeds exhibit either faster (i.e., higher relative offspring mass leading to shorter lactation length, and thus shorter interbirth interval) or slower life histories and that an early AP and a heavy relative offspring mass co‐evolved into a comparatively fast life history strategy. On the other hand, AP was positively related to lactation length: A later start of reproduction was associated with a longer lactation length. Consequently, variation in AP in pinnipeds seems to be affected by an interplay between costs and benefits of early reproduction mediated by relative investment into the single offspring via relative birth mass and lactation length.

## 1 INTRODUCTION

The age at which an organism starts to reproduce (age at primiparity, hereafter AP) is considered to be a crucial life history trait with a profound influence on fitness and life history evolution (Brommer, Pietiainen, & Kolunen, 1998; Brooke, Copas, Gylee, & Krüger, 2004; Charnov, 1997; Cole, 1954; Desprez et al., 2014; Fay, Barbraud, Delord, & Weimerskirch, 2016; Lewontin, 1965; Mourocq et al., 2016; Prevot‐Julliard, Henttonen, Yoccoz, & Stenseth, 1999; Roff, 1992; Stearns, 1992), population ecology (Krüger, 2005) and is also highly relevant in the context of conservation (Kindsvater, Mangel, Reynolds, & Dulvy, 2016; Mourocq et al., 2016). The benefits of an early start to reproduction include an increased probability of realizing reproduction, hence an increased fitness compared to a later start (Cole, 1954; McGraw & Caswell, 1996; Oli, Hepp, & Kennamer, 2002). However, delayed maturity might be favoured if the costs of early reproduction, in terms of reduced survival, future reproduction, or somatic growth, outweigh the benefits (Cody, 1971; Pyle, Nur, Sydeman, & Emslie, 1997; Stearns, 1989; Tavecchia, Pradel, Boy, Johnson, & Cezilly, 2001; Weimerskirch, 1992). The fitness consequences of this delayed maturity have received considerable theoretical (Caswell & Hastings, 1980; Charnov, 1997; Stearns & Koella, 1986) and empirical attention (Brommer et al., 1998; Desprez et al., 2014; Fay et al., 2016; Lunn, Boyd, & Croxall, 1994; McGraw & Caswell, 1996; Oli et al., 2002). This trade‐off should lead to the evolution of an optimal AP (Roff, 1992; Stearns, 1992). Indeed, in many long‐lived, iteroparous organisms, individuals commonly do not start breeding as early as they physiologically can (Fisher, 1975; Nielsen & Drachmann, 2003; Pyle et al., 1997; Weimerskirch, 1992). This has frequently been interpreted as evidence that the costs of early breeding might be higher than the benefits (Pyle et al., 1997; Weimerskirch, 1992).

Comparative analyses have largely failed to find ecological correlates of AP. Early efforts by Harvey and Clutton‐Brock (1985) and Wootton (1987) collated AP data for several hundred mammal species and found very small effects of ecology but large phylogenetic effects. Likewise, Gaillard et al. (1989) found a strong phylogenetic effect across bird taxa. General insights, however, have been notoriously difficult to come by with the one exception that phylogeny seems to be of major importance, explaining the large differences in AP seen between major taxa (Gaillard et al., 1989; Wootton, 1987). As a consequence, comparative approaches have subsequently been rarely used in the study of variation in AP (Paemelaere & Dobson, 2011; but see Mourocq et al., 2016), with heavy emphasis on intraspecific variation (Desprez et al., 2014; Fay et al., 2016; Krüger, 2005; Oli et al., 2002). Part of the problem might be that rather diverse taxonomic groups have traditionally been lumped in analyses, whereas Felsenstein (1985) already advocated the use of taxonomically well‐defined, monophyletic groups for comparative analyses. Indeed, Prothero (1993) found substantial variation in the slope of adult life span regressed on AP across mammalian taxa with no clear ecological or life history explanation.

Pinnipeds (seals, sea lions, and walruses) are therefore uniquely suited to unravel the causal relationships between ecological and evolutionary variables and AP. Although this monophyletic group comprises only 35 species (34 extant and one recently extinct) (Berta & Churchill, 2012), these vary greatly in ecology and life history but crucially, not in litter size, as they are all monotocous. This leaves offspring birth mass and lactation length as key reproductive traits that could be co‐evolving with AP in pinnipeds as offspring number is fixed at one per breeding event. In a number of pinniped species, offspring birth mass has indeed been shown to be variable and to covary with maternal mass and age as well as with environmental conditions and juvenile survival (Arnbom, Fedak, & Rothery, 1994; Costa, Trillmich, & Croxall, 1988; Forcada & Hoffman, 2014; Kraus et al., 2013; Lavigne & Kovacs, 1988; Mueller, Porschmann, Wolf, & Trillmich, 2011; Trillmich & Wolf, 2008). Maternal mass and developmental state in turn, have been shown to positively covary with AP in some species (McDonald, Goebel, Crocker, & Costa, 2012; McDonald, Goebel, Crocker, & Costa, 2012b; Reiter & LeBoeuf, 1991). Especially in pinniped species that rely on their energy reserves during breeding (capital breeders), these correlations are expected to be stronger than in species which rely on foraging during breeding (income breeders) (Boyd, 2000). Charnov and Downhower (1995) modeled the relationship between litter size and offspring birth mass variation and found strong support for an inverse relationship; hence we can expect large variation in offspring birth mass when litter size is small. A wealth of information is also available for pinnipeds, including ecological (Caro, Stankowich, Mesnick, Costa, & Beeman, 2012; Krüger, Wolf, Jonker, Hoffman, & Trillmich, 2014) and life history (Fitzpatrick, Almbro, Gonzalez‐Voyer, Hamada, et al., 2012; Fitzpatrick, Almbro, Gonzalez‐Voyer, Kolm, & Simmons, 2012; Trillmich, 1996) variables that can be used to test whether there are significant correlates of AP.

Based on this theoretical prediction of large variation in offspring mass at birth for small litter sizes and the expected trade‐off between AP and offspring mass at birth, we expect positive co‐evolution between offspring mass at birth and AP across pinnipeds. As lactation length could be another crucial variable shaping the pace of life history and as these traits are predicted to be coupled in a so‐called pace‐of‐life syndrome (Reale et al., 2010), we expect that AP and lactation length positively co‐evolve. Thus, if offspring number is fixed at one per breeding event, we could expect that the later a pinniped species starts reproduction, the larger the parental investment in form of offspring mass at birth and lactation length. However, a large relative offspring birth mass might render a long lactation period unnecessary due to the developmental stage of the offspring. Hence, we might at the same time also expect a negative association between relative offspring birth mass and lactation length. This in turn might lead to a decrease of the interbirth interval and hence an increase in the number of reproductive events over a lifetime which is in line with a fast life history. If this argument holds, a fast life history strategy in pinnipeds would then entail a short AP, a large relative offspring birth mass and a short lactation period, a combination of trait correlations which is not predicted by classic life history theory (Stearns, 1992).

We also predict that interspecific variation in AP correlates positively with life span, as predicted by the life history paradigm that the pace of life fundamentally affects reproductive timing (Charlesworth, 1994; Paemelaere & Dobson, 2011; Roff, 1992; Stearns, 1992). In other words, we expect a trade‐off between early breeding and life span. An early start to reproduction lowers the chance of dying without offspring but possibly lowers also the number of reproductive events over a life span as this might shorten the total life span. However, starting later with reproduction may allow pinniped species to have more reproductive events or healthier offspring or to provide better care, however a risk of dying before starting reproduction (Stearns, 1992).

## 2 MATERIALS AND METHODS

### 2.1 Data collection

We collated available data on all 34 currently recognized, extant, and one recently extinct pinniped species from the literature (Caro et al., 2012; Ferguson & Higdon, 2006; Krüger et al., 2014; Lindenfors, Tullberg, & Biuw, 2002), including the following continuous variables: mean male body mass, mean female body mass, the resulting sexual size dimorphism (that is the ratio of mean male to mean female body mass), average harem size, mean birth mass, relative birth mass (mean pup birth mass as a proportion of mean adult female mass), the average length of the breeding season in days, the average length of the lactation period in days, mean breeding latitude in degrees from the equator and maximum life span in years (Tacutu et al., 2018). Maximum life span in years should be considered with some caution as it is likely to be the most unreliably measured variable. Furthermore, in order to consider the tight allometric scaling relationship between life history traits and body size (Paemelaere & Dobson, 2011), we also used partial correlation to control for body size (Baba, Shibata, & Sibuya, 2004). Mean AP in years for females was obtained from the recently published Encyclopedia of Marine Mammals (Würsig, Thewissen, & Kovacs, 2017), the Handbook of the Mammals of the World (Wilson & Mittermeier, 2014), and the AnAge database (Tacutu et al., 2018). We realize that age at first reproduction (just as lactation length) varies substantially intraspecifically, often related to differences between populations within a species, population density, and changes in food abundance. Moreover, intraspecifically, primiparous females generally produce smaller offspring than older females. We are also aware that lumping of intraspecific variation into a species‐specific value is a general shortcoming of comparative analyses. Nevertheless, we believe that the mean age at first reproduction—as determined from population studies—represents a life history trait that can be used to characterize a species' reproductive strategy in comparisons with other species in the same group.

For species with different AP data from these sources, preference was given to the Encyclopedia as it was the most recent and complete dataset, followed by the Handbook and finally the AnAge database. If no data could be found for a species from all three sources, we used the value for the sister taxon. Raw data for all variables with large variation or skew were log‐transformed before analyses. Significant positive correlations were found between the AP values of the different datasets (Encyclopedia:Handbook, *r* = 0.585, *df* = 23, *p* = 0.002; Handbook:AnAge, *r* = 0.399, *df* = 23, *p* = 0.048; Encyclopedia:AnAge, *r* = 0.420, *df* = 23, *p* = 0.037).

### 2.2 Phylogenetic comparative data analyses

In order to identify traits with correlated evolution, we analyzed our data using the phylogenetic generalized least square (PGLS) method (Freckleton, 2009; Revell, 2010), based on the complete phylogeny of all pinniped species of Higdon, Bininda‐Emonds, Beck, and Ferguson (2007) with the Galapagos sea lion (*Zalophus wollebaeki*) added with a split from the California sea lion at 2.3 million years ago (Wolf, Tautz, & Trillmich, 2007). The PGLS method also estimated the internal branch length (*λ*) which revealed the strength of the phylogenetic signal. Raw data with large variation or skew were log‐transformed. Statistical significance (*p* < 0.05) was determined using *t* tests.

## 3 RESULTS

Mean AP varies tremendously in pinnipeds (Appendix 1), from 3 years in the hooded seal (*Cystophora cristata*) to 10 years in the walrus (*Odobenus rosmarus*). There is no significant difference in the mean or variance of ages at primiparity between eared and true seals (*F*_1,32_ = 0.161, *p* = 0.691), regardless which data for AP are used; hence this deep phylogenetic split cannot explain the variation in age of primiparity observed.

**Figure 1:**
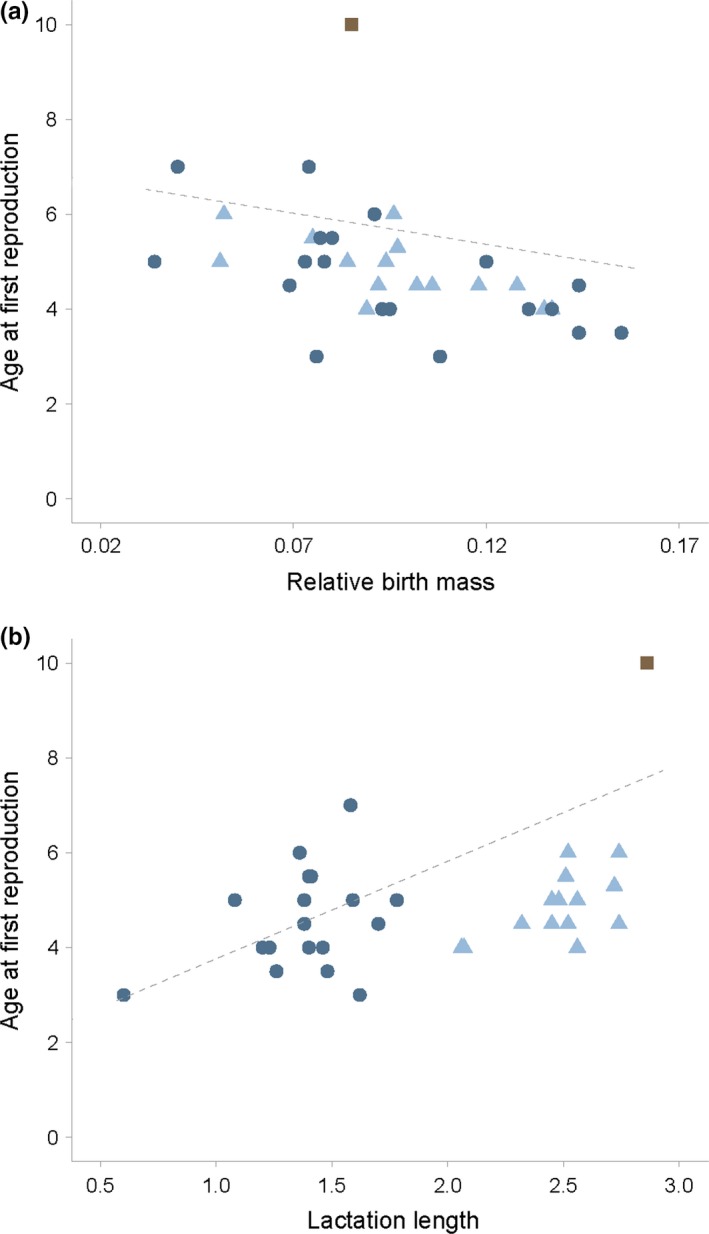
Scatterplots of relative birth mass against age at primiparity (a) and of lactation length against age at primiparity (b). Odobenidae;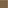, Otariidae;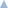, Phocidae;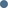, best‐fit line;
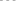

Age at primiparity is significantly positively correlated with lactation length (Figure 1; estimate ± *SE* = 2.055 ± 0.346, *F* = 35.31, *p* < 0.001, *λ* = 1.000), while it is significantly negatively correlated with relative birth mass (Figure 1; estimate ± *SE* = −13.130 ± 4.297, *F* = 9.334, *p* = 0.004, *λ* = 0.962). All other variables had no influence on AP (Table 1). Furthermore, lactation length was significantly negatively correlated with relative birth mass (estimate ± *SE* = −3.463 ± 1.264, *F* = 7.503, *p* = 0.010, *λ* = 0.930). The high lambda values indicate a strong phylogenetic signal, that is closely related species tend to resemble each other more. This is quite likely due to the three pinniped families combined in the same tree.

**Table 1:** Phylogenetic generalized least square results between age at primiparity and explanatory variables across species with standard error and lambda

Variable	Estimates ± *SE*, *λ*
Female mass	0.752 (0.731), 0.940
Male mass	−0.197 (0.788), 1.000
Sexual size dimorphism	−0.150 (0.172), 0.961
Harem size	−0.006 (0.020), 0.953
Birth mass	0.013 (0.023), 0.943
Relative birth mass	−13.129 (4.297)**, 0.962
Breeding season length	−0.236 (0.631), 0.949
Lactation length	2.055 (0.346)***, 1.000
Breeding latitude	−0.008 (0.009), 0.959
Lifespan	0.037 (0.027), 0.975

Note

Significant values are indicated by asterisk:

* *p* < 0.05,

** *p* < 0.01,

*** *p* < 0.001.

## 4 DISCUSSION

We found that across pinniped species, only two of our variables (relative birth mass and lactation length) were significantly associated with interspecific variation in AP. This paucity of correlates is in line with earlier analyses on much broader taxonomic scales that found surprisingly few correlates (Gaillard et al., 1989; Wootton, 1987). Not even female body mass was positively associated with AP.

Two significant evolutionary correlates of AP were found—relative birth mass and lactation length. An earlier start of the reproductive career was associated with heavy pups relative to the female's mean body mass and a late start was associated with light pups relative to the female's mean body mass. This indicates that a large relative offspring mass might be a way to shorten lactation length and hence also the interbirth interval. Thereby pinnipeds could shift their life history toward the faster end of the pace of life syndrome. In line with this reasoning, we also found a negative relationship between relative offspring birth mass and lactation length. This supports our argument that a high relative birth mass might have facilitated the evolutionary shortening of lactation length and thereby sped up the life history.

In contrast, an early start of the reproductive career was associated with short lactation times while a late start was associated with long lactation times. The negative correlation between relative birth mass and AP contradicts our prediction about a trade‐off between AP and offspring mass. However, it is in line with a key result of Paemelaere and Dobson (2011) who found a significant negative association between litter size and AP in carnivorous mammals. As litter size is fixed at one in the monotocous pinnipeds, all that can be varied is relative offspring mass, which seems to have negatively co‐evolved with AP.

Our second prediction was also not supported by our results. We found no significant positive association between AP and life span across species. As a result, we found little support for a clear evolutionary link between AP and life span in pinnipeds. While this could well be due to data inaccuracies, as maximum life span is a notoriously hard to measure variable, particularly in marine mammals, it could also suggest a lack of trade‐off between early breeding and life span. Life span could be limited by factors other than reproductive stress, or species might compensate the cost of early AP with longer interbirth intervals thereby having the same reproductive cost as the ones with late AP and shorter interbirth intervals at the end. A nonsignificant association between variation in AP and life span has also been reported by Paemelaere and Dobson (2011) for carnivorous mammals, however, a recent analysis in birds found a significant positive relationship (Mourocq et al., 2016).

Another relationship we found was between AP and lactation length, which was significantly positive. This means as female pinnipeds mature later, their lactation lengths increase. This positive association between AP and lactation length is predicted by classic life history (Stearns, 1992) and the pace‐of‐life syndrome (Reale et al., 2010). As the trade‐off between offspring mass and offspring number is fixed in pinnipeds, the evolution of life histories in this taxon might have led to a trade‐off between offspring mass and the time it takes mothers to wean these offspring. Intraspecifically, this trade‐off has been shown, for example, in Galápagos fur seals (Trillmich & Dellinger, 1991; Trillmich & Limberger, 1985) or Australian sea lions (Costa et al., 1988; Fowler, Costa, Arnould, Gales, & Kuhn, 2006; Fowler, Costa, & Arnould, 2007; Fowler, Costa, Arnould, Gales, & Burns, 2007). If prey availability was low, pups were born lighter and lactation length was longer, which then lead to a longer interbirth interval.

In conclusion, this study has shown that variation in AP in pinnipeds very likely co‐evolved with relative birth mass, so that an early start is associated with large relative offspring mass which can then be weaned early, giving rise to a fast–slow continuum of pinniped life histories. It would be informative to compare the results of this study with two other large monotocous taxa, the mammalian bats (Chiroptera) and the avian tubenoses (Procellariiformes), with the potential caveat that the need to fly might change the selective landscape completely. Indeed, Prothero (1993) found the relationship between adult age and AP to be highly anomalous in bats as it was not related to body mass.

## CONFLICT OF INTEREST

None declared.

## AUTHOR CONTRIBUTIONS

S.K and O.K conceived and designed the study, S.K and O.K collected the data, O.K. and S.K analyzed the data, O.K and S.K wrote the draft and all authors contributed and critically edited the final manuscript.

## Data Availability

The datasets generated during and/or analyzed during the current study are available at Dryad Digital Repository DOI: https://doi.org/10.5061/dryad.8s715qp.

## APPENDIX 1

Raw data on age at primiparity (AP) in each extant species of pinniped from the recently published Encyclopedia of Marine Mammals (Würsig et al., 2017), the Handbook of the Mammals of the World (Wilson & Mittermeier, 2014), and the AnAge database (Tacutu et al., 2018).

**Table:**

Taxon	AP encyclopedia	AP handbook	AP AnAge
*Odobenus rosmarus rosmarus*	10.0	8.0	4.8
*Callorhinus ursinus*	4.0	4.0	3.0
*Neophoca cinerea*	5.3	5.3	3.0
*Otaria flavescens (byronia)*	4.5	4.0	4.0
*Arctocephalus pusillus*	4.5	4.5	3.5
*Phocarctos hookeri*	5.0	3.5	
*Arctocephalus forsteri*	5.0	5.0	
*Arctocephalus australis*	4.0	3.0	
*Arctocephalus galapagoensis*	4.5	4.0	
*Arctocephalus gazella*	4.0	3.0	3.5
*Arctocephalus tropicalis*	5.0	5.0	
*Arctocephalus philippii*			
*Arctocephalus townsendi*			
*Eumetopias jubatus*	5.5	4.5	4.9
*Zalophus californianus*	6.0	4.5	3.0
*Zalophus wollebaeki*	6.0	4.5	
*Erignathus barbatus*	5.0	4.5	4.7
*Cystophora cristata*	3.0	3.5	3.0
*Phoca hispida*	5.0	5.0	5.0
*Phoca sibirica*	5.0	4.5	5.8
*Halichoerus grypus*	4.0	4.0	4.0
*Phoca caspica*	6.0	5.0	5.5
*Phoca largha*	3.5	4.5	3.5
*Phoca vitulina*	4.5	4.5	3.0
*Histriophoca fasciata*	4.0	4.0	2.9
*Phoca groenlandica*	5.0	5.5	4.5
*Lobodon carcinophagus*	4.0
*Ommatophoca rossi*	3.5	3.0
*Hydrurga leptonyx*	4.0	4.5	3.0
*Leptonychotes weddellii*	4.5	7.0	3.7
*Mirounga angustirostris*	5.5	3.0	3.0
*Mirounga leonina*	5.5	3.0	2.9
*Monachus monachus*	3.0	4.0
*Monachus schauinslandi*	7.0	5.0
*Monachus tropicalis*			
